# Culturable endophytic bacteria associated with medicinal plant *Ferula songorica*: molecular phylogeny, distribution and screening for industrially important traits

**DOI:** 10.1007/s13205-016-0522-7

**Published:** 2016-09-28

**Authors:** Yong-Hong Liu, Jian-Wei Guo, Nimaichand Salam, Li Li, Yong-Guang Zhang, Jian Han, Osama Abdalla Mohamad, Wen-Jun Li

**Affiliations:** 1 Key Laboratory of Biogeography and Bioresource in Arid Land, Xinjiang Institute of Ecology and Geography, Chinese Academy of Sciences, Urümqi, 830011 Xinjiang People’s Republic of China; 2 State Key Laboratory of Biocontrol and Guangdong Provincial Key Laboratory of Plant Resources, School of Life Sciences, Sun Yat-Sen University, Guangzhou, 510275 People’s Republic of China; 3 Key Laboratory of Crops with High Quality and Efficient Cultivation and Security Control, Yunnan Higher Education Institutions, Honghe University, Mengzi, 661100 People’s Republic of China; 4 Environmental Science Department, Institute for Post Graduate of Environment Study, Arish University, Arish, 45511 Egypt; 5 University of Chinese Academy of Sciences, Beijing, 10049 People’s Republic of China

**Keywords:** *Ferula songorica*, Endophytes, Diversity, Growth promotion, Enzyme

## Abstract

Xinjiang, a region of high salinity and drought, is a host to many arid and semi-arid plants. Many of these plants including *Ferula* spp. have indigenous pharmaceutical histories. As many of the medicinal properties of plants are in tandem with the associated microorganisms residing within the plant tissues, it is advisable to explore the endophytic potential of such plants. In the present study, diversity of culturable bacteria isolated from medicinal plants *Ferula songorica* collected from Hebukesaier, Xinjiang were analyzed. A total of 170 endophytic bacteria belonging to three phyla, 15 orders, 20 families and 27 genera were isolated and characterized by 16S rRNA gene sequencing. The phylum Actinobacteria constitutes a major portion of the endophytic microbes isolated from the plant *Ferula songorica* (107 isolates). Overall endophytic species richness of the sample was 58 taxa while the sample has statistical values of 4.02, 0.97, 0.65 and 16.55 with Shannon’s, Simpson, Species evenness and Margalef, respectively. Root tissues were found to be more suitable host for endophytes as compared to leaf and stem tissues. Among these endophytic strains, 88 % can grow on nitrogen-free media, 19 % solubilize phosphate, while 26 and 40 % are positive for production of protease and cellulase, respectively. The results confirm that the medicinal plant *Ferula songorica* represents an extremely rich reservoir for the isolation of diverged bacteria with potential for growth promoting factors and biologically active compounds including enzymes.

## Introduction

*Ferula* of the family Umbelliferae is a genus of about 180 species of flowering plants and are native to Mediterranean, central Asia and its adjacent areas (Pimenov and Leonov 2004). Twenty-six of these species are found in Xinjiang Uyghur Autonomous Region (People’s Republic of China). Among them, the variety *Ferula songorica* are distributed in several places of Xinjiang including Hebukesaier, Emin county, Toli county, Yumin county and Tahcheng. Traditionally, they are being utilized for treatments of digestive disorders, rheumatism, headache, dizziness, toothache, etc. (Sun et al. 2013). Excessive excavation for medicinal purposes and man-made destruction during the recent years have, however, severely reduced the number of wild *Ferula* plants, and have even led to extinction of some varieties. It has now come to a stage that it is almost impractical to conserve *Ferula* plants through artificial cultivation, and therefore, a proper mechanism should be enforced to protect these precious medicinal resources including *Ferula songorica*.

One such mechanism is to explore the biotechnological potential of the microbial communities residing within the floral resources that are commonly referred to as endophytes. Endophytes have been proven to show positive effects on the host plants by serving as growth promoter, insect and pest repellents, antimicrobial agents against plant pathogens and stress modulators (Ryan et al. 2008; Staniek et al. 2008; Nagabhyru et al. 2013; Rai et al. 2014). A deeper look on endophytes also revealed that they are a source of various bioactive substances that find importance to ecology, medicine, pathology and agriculture (Wang et al. 2008). For example, an endophytic *Promicromonospora* sp. isolated from *Artemisia annua* in Yunnan was found to be proteinase and cellulase-producer (Li 2010). Endophytic bacterial strains *Isoptericola rhizophila*, *Nitratireductor shengliensis*, *Paenibacillus lautus* and *Staphylococcus xylosus* associated with halophytes from Xinjiang possess phosphate-solubilizing activities (Huang et al. 2010; Wang 2015).

Of the myriad ecosystems on earth, those with the greatest general biodiversity seem also to have the greatest number and the greatest diversity of endophytes (Strobel et al. 2004). Till date, only a few plants have ever been completely studied in relation to their endophytic biology. The necessity of finding new and beneficial endophytic microorganisms among the wide diversity of plants in different ecosystems is hence considerable. Xinjiang, a typical arid environment, is located in the border areas of northwest China and falls in the center of the Eurasian region. Endophytes adapting this special environment are likely to produce special metabolites. It is, therefore, necessary to explore these special endophytic resources of arid and extremely arid desert habitats. The present study involved the isolation of endophytes associated with medicinal plant *Ferula songorica*, analyzed the species richness and distribution pattern among the different tissues of the plant. In addition, the plant growth promoting traits and their ability to produce industrial enzymes were studied. The present study, in a small way, will help in conserving the *Ferula* plant from mass-scale excavation by exploiting the associated endophytes instead of the plant itself.

## Materials and methods

### Sample collection and surface sterilization

Plant samples of *Ferula songorica* were collected from an alluvial fan located at Hebukesaier, Xinjiang (46°52′N, 85°55′E; 1443 m above sea level) on June 8, 2015. Three samples were randomly collected, each separated by at least 500 m apart. The plant samples were transported to laboratory in sterile plastic bags and processed within 24 h. The samples were washed in running tap water to remove the clays on the surface of plant tissue and checked for disease symptoms or superficial damage. Symptom-free plant samples were then washed in a water bath sonicator repeatedly until the water become clear. After proper washing, the samples were separated into leaves, stems and roots. Each tissue were separately surface-sterilized by stepwise washing in 75 % ethanol for 1 min, sodium hypochlorite solution for 8 min followed by five rinses in sterile distilled water. Two experiments were carried out to check the effectiveness of sterilization procedures. First, the sterilized-surface tissue was imprinted directly onto yeast extract-malt extract agar (ISP 2), incubated at 30 °C, and checked for microbial growth. Second, the sterile distilled water used in the final rinse was plated onto ISP 2 agar plate and incubated at 30 °C. If no microbial growth occurred on the surface of the medium, the sterilization was considered complete.

## Isolation of endophytic bacteria

Samples were air-dried for 2 days at room temperature and were aseptically homogenized by sterilized commercial blender. Tissues homogenates were then pretreated by one of the following methods:

### Method 1

Directly placed on the selective isolation media (Table 1), and incubated at 30 °C for 2–8 weeks.

**Table 1:** Compositions of the nine different media used for the isolation of endophytic bacteria from plant samples

Medium	Composition (g/L)	References
M1	Sodium propionate, 2; l-asparagine, 1; (NH_4_)_2_SO_4_, 0.1; KCl, 0.1; MgSO_4_·7H_2_O, 30; FeSO_4_·7H_2_O, 0.05; agar, 15	Wang (2015)
M2	Yeast, 0.25; K_2_HPO_4_, 0.5; NaCl, 30; agar, 15	Wang (2015)
M3	Sodium propionate, 2; l-asparagine, 1; NH_4_NO_3_, 0.1; KCl, 0.1; MgSO_4_·7H_2_O, 0.05; FeSO_4_·7H_2_O, 0.05; NaCl, 30; agar, 15	Li (2010)
M4	Cellulose, 2.5; sodium pyruvate, 2; KNO_3_, 0.25; proline, 1; MgSO_4_·7H_2_O, 0.2; K_2_HPO_4_, 0.2; CaCl_2_, 0.5; FeSO_4_·7H_2_O, 10 mg; NaCl, 30; agar, 15	Modified from Qin et al. (2009)
M5	Sodium oxalate, 2; Casein hydrolysate 0.5; KH_2_PO_4_, 0.3; Na_2_HPO_4_·12H_2_O, 0.5; NaCl, 30; ZnSO_4_·7H_2_O, 0.02; CaCl_2_, 0.5; agar, 15	Wang (2015)
M6 (ISP 5)	Glycerol, 10; l-asparagine, 1; NaCl, 30; K_2_HPO_4_, 1; agar, 15	Shirling and Gottlieb (1966)
M7	Added to Sodium propionate, 1; l-asparagine, 0.2; KH_2_PO_4_, 0.9; K_2_HPO_4_, 0.6; MgSO_4_·7H_2_O, 0.1; CaCl_2_·2H_2_O, 0.2; NaCl, 30; KCl, 0.3; FeSO_4_·7H_2_O, 0.001; agar, 15	Modified from Qin et al. (2009)
M8	Sodium propionate, 2; Arginine, 1; NaCl, 30; MgSO_4_·7H_2_O, 1; KH_2_PO_4_, 0.1; FeSO_4_·7H_2_O, 0.05; agar, 15	Wang (2015)
M9 (ISP 4)	Soluble starch, 20; KNO_3_, 1; K_2_HPO_4_, 0.5; MgSO_4_·7H_2_O, 0.5; NaCl, 0.5; FeSO_4_·7H_2_O, 0.01; agar, 15	Shirling and Gottlieb (1966)

### Method 2

About 1 g tissue homogenates was taken to a sterilized tube, added with 9 ml sterile water and thoroughly mixed to give a tissue suspension. The tissue suspension was diluted to a concentration of 10^−2^ and 10^−3^ followed by plating of 40 µl each of the diluted suspension onto the isolation media. The isolation plates were incubated at 30 °C for 3–12 weeks.

All experiments were done in duplicate. Pure cultures obtained in the isolation media were grown and maintained in ISP 2 agar.

### Genomic DNA extraction

Enzymatic hydrolysis was used to extract genomic DNAs of all bacteria. About 50 mg of the freshly grown culture was taken in an autoclaved 1.5 ml Eppendorf tube. To the culture, 480 µl TE buffer (1×) and 20 µl lysozyme solution (2 mg/ml) were added. The bacterial suspension was thoroughly mixed and incubated for 2 h under shaking conditions (160 rpm, 37 °C). The mixture was treated with 50 µl SDS solution (20 %, w/v) and 5 μl Proteinase K solution (20 µg/ml), and kept on a water bath (55 °C, 1 h). DNA was then extracted twice with phenol–chloroform–isoamyl alcohol (25:24:1 v/v/v), followed by precipitation with 80 µl sodium acetate (3 mol/l, pH 4.8–5.2) and 800 µl absolute ethanol. The resulting DNA precipitate was centrifuged at 4 °C (12,000 rpm, 10 min), washed with 70 % ethanol, and then air-dried. The extracted DNA was resuspended in 40 μl sterile Milli-Q water and stored at −20 °C for further use.

### Sequencing and diversity analysis

The isolates were subjected to 16S rRNA gene sequence analysis for identification at the genus level. Amplification of the 16S rRNA gene was done using the primer pair PA-PB (PA: 5′-CAGAGTTTGATCCTGGCT-3′; PB: 5′-AGGAGGTGATCCAGCCGCA-3′) procured from Sangon Biotech (Shanghai, China). Amplified PCR products were purified and sequenced by Sangon Biotech (Shanghai). The sequences obtained were identified using the EzTaxon-e server database. A sequence similarity of less than 98.65 % from the published database was considered to be a novel strain (Kim et al. 2014). These sequences were then aligned using ClustalX v.2.1 (Larkin et al. 2007). Phylogenetic dendrogram based on the 16S rRNA gene sequences was then generated using neighbor-joining method from MEGA 5.1 software package (Tamura et al. 2011).

Diversity of the endophytes in the plant samples was analyzed using the software package PAST 2.03 with relation to the different statistical parameters (Species richness, Shannon’s index, Simpson index, Species evenness and Margalef index) (Whittaker 1972; Hammer et al. 2001; Huang et al. 2013).

### Growth promotion and enzyme activity

The endophytic strains were tested for growth promoting traits and production of protease and cellulase. For growth promoting traits, the ability of the strains to solubilize phosphate and fix nitrogen were measured as described by Hu et al. (2012) and Wang (2015). Strain was considered to be able to fix nitrogen if it exhibit growth on both Ashby medium and nitrogen-free culture medium (Hopebio Company, Qingdao, China) (Sen and Sen 1965). Production of protease and cellulase by the endophytic strains were measured as described by Li (2011).

## Results

### Effectiveness of surface sterilization

No microbial growth was observed after 15 days of incubation at 30 °C when either the sterilized-surface tissue were directly imprinted or the water of final rinse were plated on ISP 2 agar. This indicated that the five-step surface sterilization protocol was effective at inhibiting the growth of epiphytic bacteria. Thus, the subsequent isolates can be considered to be true endophytic bacteria.

### Diversity of endophytic bacteria

**Fig. 1:**
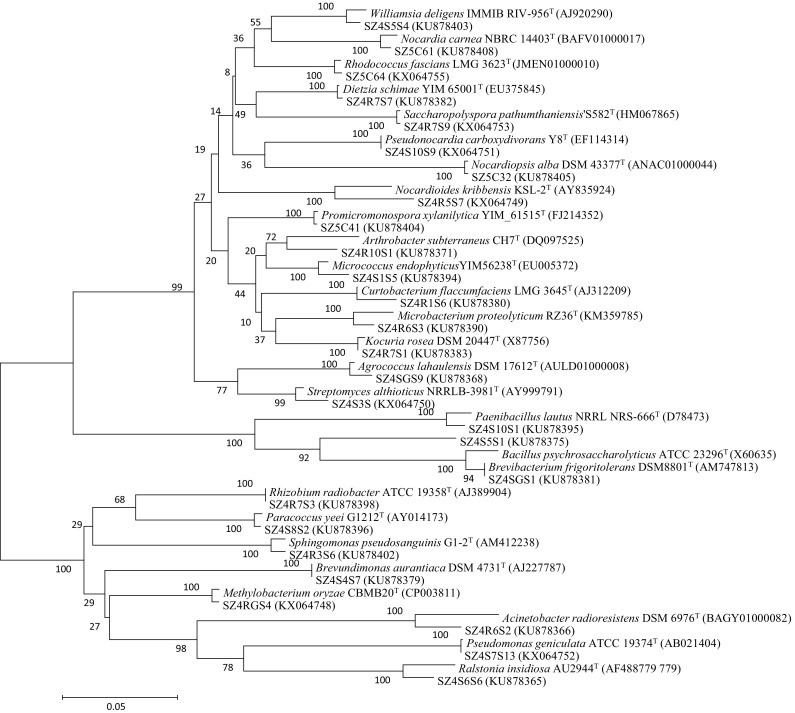
Neighbour-joining phylogenetic tree based on partial 16S rRNA gene sequences of the representative strains. *Numbers* in the branches represent the bootstrapping percentage that supports the branch with 1000 bootstrap replications

The endophytic bacteria isolated in this study displayed considerable diversity. A total of 170 endophytic bacterial isolates representing 58 taxa were isolated from symptom-free, surface sterilized tissues of Ferula songorica. Maximum isolates were obtained from roots (88 isolates) while the rest (82) from either leaves or stems. The distribution of the bacterial strains isolated from Ferula songorica among the different phyla are listed in Table 2. The phylum *Actinobacteria* dominated the endophytic bacterial community in *Ferula songorica*, representing 62.9 % of the total isolates. The remaining isolates were represented by the phyla *Proteobacteria* and *Firmicutes* which constitute 23.5 and 13.5 %, respectively. The most predominantly isolated genera among these isolates were *Brevundimonas* (23 isolates)*, Sphingomonas* and *Bacillus* (22 each). While the frequencies of certain genera *Microbacterium, Agrococcus, Arthrobacter, Kocuria, Micrococcus, Promicromonospora, Nocardiopsis, Williamsia, Streptomyces, Pseudonocardia, Dietzia, Acinetobacter, Methylobacterium, Rhizobium* and *Ralstonia* falls between 1 and 8 %, several isolates were isolated as single strain and these include the genera *Curtobacterium, Brevibacterium, Rhodococcus, Nocardia, Saccharopolyspora, Nocardioides, Paracoccus* and *Paenibacillus*. Fig. 1 depicts the dendrogram based on the 16S rRNA gene sequences representing a randomly selected strain from each genera associated with *Ferula songorica*. Among the different isolates, strain SZ4R5S7 exhibited 97.1 % 16S rRNA gene sequence similarity with *Nocardioides salsibiostraticola* PAMC 26527^T^ indicating that the strain could be new member of the genus *Nocardioides*.

**Table 2:** Distribution of strains isolated from *Ferula songorica*

Phyla (3)	Orders (15)	Families (20)	Genera (27)	Species	Strains
				(58)	(170)
*Actinobacteria*	*Micrococcales*	*Microbacteriaceae*	*Microbacterium*	3	9
			*Curtobacterium*	1	1
			*Agrococcus*	1	3
		*Micrococcaceae*	*Arthrobacter*	4	5
			*Kocuria*	3	11
			*Micrococcus*	3	5
		*Brevibacteriaceae*	*Brevibacterium*	1	1
		*Promicromonosporaceae*	*Promicromonospora*	2	5
	*Streptosporangiales*	*Nocardiopsaceae*	*Nocardiopsis*	3	8
	*Corynebacteriales*	*Nocardiaceae*	*Williamsia*	1	4
			*Rhodococcus*	1	1
			*Nocardia*	1	1
	*Streptomycetaceae*	*Streptomycetales*	*Streptomyces*	5	7
	*Pseudonocardiales*	*Pseudonocardiaceae*	*Pseudonocardia*	2	2
			*Saccharopolyspora*	1	1
	*Propionibacteriales*	*Nocardioidaceae*	*Nocardioides*	1	1
	*Corynebacteriales*	*Dietziaceae*	*Dietzia*	2	2
*Proteobacteria*	*Sphingomonadales*	*Sphingomonadaceae*	*Sphingomonas*	4	22
	*Pseudomonadales*	*Moraxellaceae*	*Acinetobacter*	2	7
	*Gammaproteobacteria*	*Xanthomonadales*	*Pseudomonas*	1	1
	*Rhizobiales*	*Methylobacteriaceae*	*Methylobacterium*	2	7
		*Rhizobiaceae*	*Rhizobium*	1	14
	*Rhodobacterales*	*Rhodobacteraceae*	*Paracoccus*	1	1
	*Burkholderiales*	*Burkholderiaceae*	*Ralstonia*	1	5
	*Caulobacterales*	*Caulobacteraceae*	*Brevundimonas*	1	23
*Firmicutes*	*Bacillales*	*Paenibacillaceae*	*Paenibacillus*	1	1
		*Bacillaceae*	*Bacillus*	9	22

Table 3 represents the statistical values from PAST analysis indicating bacterial diversity associated with the plant *Ferula songorica*. Overall species richness of the plant sample was determined to be 58 taxa while the index values according to Shannon, Simpson, Species evenness and Margalef are 4.02, 0.97, 0.65 and 16.55, respectively. All the indices suggest a relatively high diversity of the endophytic bacterium community.

**Table 3:** Diversity of endophytic strains

	Individuals	Taxa_S	Shannon_H	Simpson	Evenness	Margalef
Leaf and stem	82	40	3.42	0.96	0.76	8.85
Root	88	43	3.9	0.97	0.79	13.62
Total	170	58	4.02	0.97	0.65	16.55

(Shannon H: variety; Simpson: dominance index; Evenness: uniformity; Margalef: abundance)

### Tissue-specificity of endophytes

**Fig. 2:**
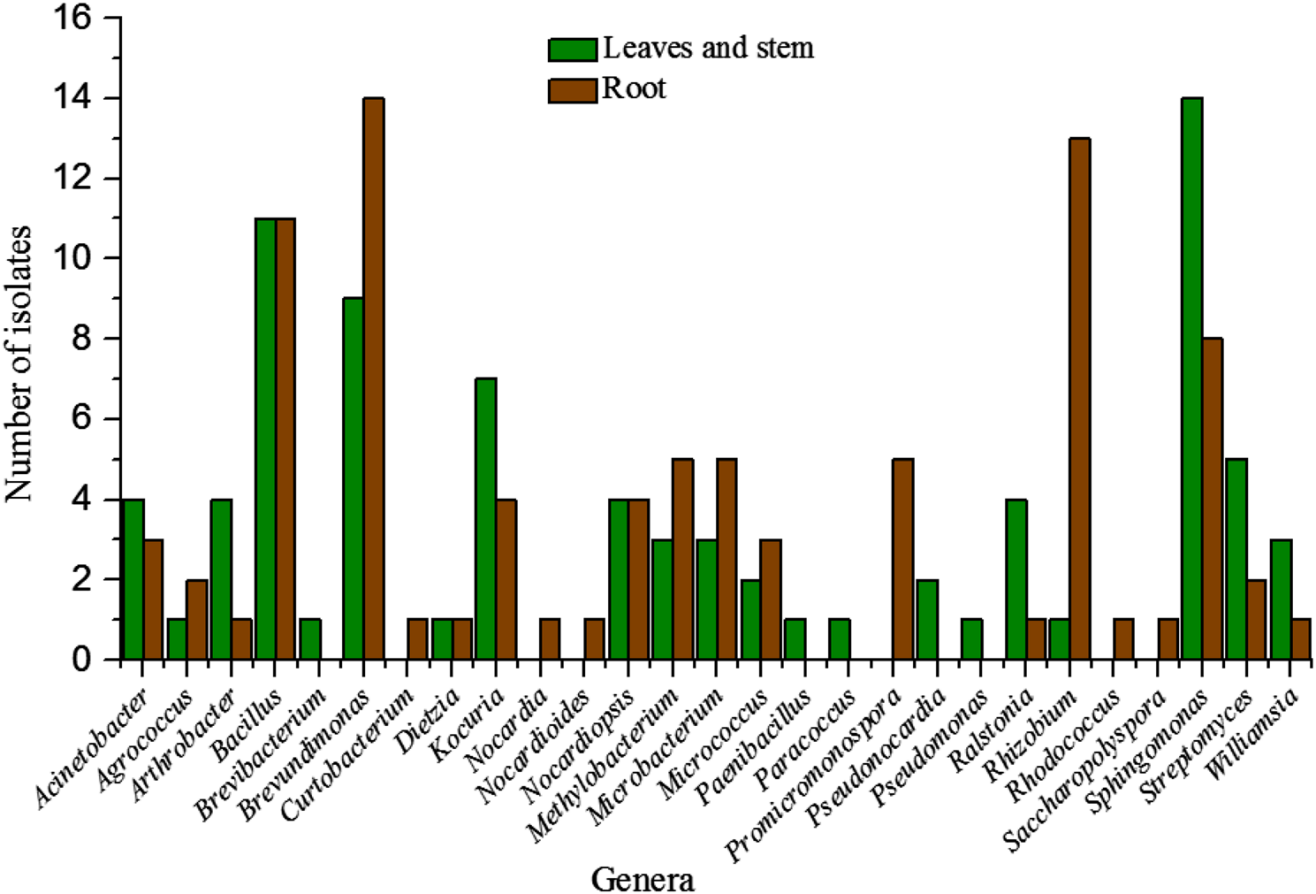
Diversity of endophytic strains from *Ferula songorica*

More isolates were obtained from roots than the other two tissues used in the current study (Table 3). Among the different tissues, the distribution of the genera *Agrococcus*, *Brevundimonas*, *Methylobacterium*, *Microbacterium*, *Micrococcus* and *Rhizobium* were higher in roots than in leaves and stems while that of *Acinetobacter*, *Arthrobacter*, *Kocuria*, *Ralstonia*, *Sphingomonas*, *Streptomyces* and *Williamsia* were more in leaves and stems than roots (Fig. 2). However, some of the genera are restricted to a particular tissue as indicated by the isolation of genera *Brevibacterium*, *Paenibacillus*, *Paracoccus*, *Pseudonocardia* and *Pseudomonas* from leaves and stems, and *Curtobacterium*, *Nocardia*, *Nocardioides*, *Promicromonospora*, *Rhodococcus* and *Saccharopolyspora* from roots. The other three genera were represented at the same rate, for instance, *Bacillus*, *Dietzia* and *Nocardiopsis* (Fig. 2). Based on the PAST analysis (Table 3) species richness was little higher in roots than leaves and stems while species evenness were relatively similar in both the type of tissues (0.79 and 0.76 for roots and ground tissues communities, respectively).

### Growth promotion and enzyme activity

Plant growth promoting ability with respect to nitrogen fixation and phosphate solubilization, and enzyme activities with respect to protease and cellulase were tested. It was found that 88 % of the strains have the capacity of fixing nitrogen while 19 % can solubilize phosphate. 26 % of the strains can produce protease while 40 % are positive for cellulase production. Table 4 list the endophytic strains that are positive for two or more of the activities tested. Strains SZ4R6S2 and SZ4S4S9 showed high ability of phosphate-solubilization followed by strain SZ4R5S4. Except for strains SZ4R7S8, SZ4S8S4 and SZ4S7S13, all strains in Table 4 could fix nitrogen. While strains SZ4S8S4, SZ4S7S13, SZ4R7S10 and SZ4R1S6 are highest protease-producers, strains SZ4S5S1, SZ4R7S10, SZ4R7S8 and SZ4SGS1 are the best cellulase-producing strains among all the endophytes isolated during the present study. Strains SZ4R7S10, SZ4R1S6 and SZ4S6S2 were positive for all the four tests, while strains SZ4S5S1, SZ4S81, SZ4SGS1, SZ4R3S8, SZ4S5S2, SZ4R5S3 and SZ4R3S7 tested positive for three of the activities tested.

**Table 4:** Characteristics and enzymatic screening of some representative strains

Strain	Closest homolog	Growth promoting activity		Enzyme activity	
		Fix N	Solubilize P	Protease	Cellulase
SZ4R5S4	*Acinetobacter pittii*	+	++	−	−
SZ4R6S2		+	+++	−	−
SZ4S5S1	*Bacillus endophyticus*	+	−	+	++
SZ4S81	*Bacillus licheniformis*	+	−	+	+
SZ4R7S10	*Bacillus safensis*	+	+	++	++
SZ4R7S8		−	−	+	+++
SZ4R3S14	*Brevundimonas aurantiaca*	+	−	−	−
SZ4S8S14		+	−	−	+
SZ4SGS1	*Brevibacterium frigoritolerans*	+	−	+	++
SZ4R1S6	*Curtobacterium flaccumfaciens*	+	+	++	+
SZ4R3S8	*Microbacterium hydrothermale*	+	+	−	+
SZ4S6S2		+	+	+	+
SZ4S5S2		+	+	−	+
SZ4R5S3		+	+	−	+
SZ4S8S16		+	−	−	+
SZ4R3S7	*Microbacterium testaceum*	+	−	+	+
SZ4S8S4	*Micrococcus aloeverae*	−	−	+++	−
SZ4S10S1	*Paenibacillus lautus*	+	−	−	−
SZ4S8S2	*Paracoccus yeei*	+	+	−	−
SZ4S7S13	*Pseudomonas geniculata*	−	−	+++	−
SZ4S7S14	*Rhizobium radiobacter*	+	−	−	−
SZ4S4S9	*Sphingomonas paucimobilis*	+	+++	−	−

The ability to solubilize P, protease and cellulase were represented by halo diameter/colony diameter (*R/r*) whereby

– (negative), *R/r* = 1.0 cm (without halo zone and no enzyme activity); + (weakly positive), 1.0 cm < *R/r* < 2.0 cm; ++ (moderately positive), 2.0 cm ≤ *R/r* < 3.0 cm; +++ (strongly positive), *R/R* ≥ 3.0 cm

The ability of fix N was recorded as “+” if strain grows on both the nitrogen growth media

## Discussion

Microorganisms are ubiquitous, abundant, and diverse in natural environments, and play important roles in a number of environmental processes. An extensive characterization of diverse population of endophytic bacteria associated with medicinal plants can provide a greater insight into the plant-endophyte interactions, bioactivity and ecological role (Bloemberg and Lugtenberg 2001). Determination of community structure in different tissues is, therefore, essential for subsequent application of bacteria in improvement of site adaptation of plants in extreme environment such as saline soils (Szymańska et al. 2016). In this study, 170 pure bacterial cultures belonging to three phyla, 15 orders, 20 families and 27 genera were isolated using two isolation procedures and nine selective media. The dominant endophytic genera in *Ferula songorica* belong to *Brevundimonas*, *Sphingomonas* and *Bacillus*. As many members of the genus *Brevundimonas* were reported from soil (Yoon et al. 2006; Kang et al. 2009; Wang et al. 2012), the predominant isolation of *Brevundimonas* during the present study may be associated with the theory that most endophytic bacteria come from the surrounding rhizosphere (Long et al. 2010). The genus *Sphingomonas* have been isolated from plant tissue and some of these species were identified as potential growth regulators of crops (Chen et al. 2014; Khan et al. 2014; Yang et al. 2014; Halo et al. 2015). *Bacillus* spp. isolated from plant tissue has high richness, and has greater potential as biocontrol agents and plant growth promoters (Kumar et al. 2012). These results were similar with the present study that endophytic bacteria have the promoting ability of nitrogen fixation and phosphate solubilization.

During the present study, more isolates were obtained from root tissues (51.8 % of all isolates) than stem and leaf (48.2 %). Statistical indices also indicate a higher diversity of endophytes in root than that in stem and leaf. These results were in consistent with that of Chen et al. (2012) and Ma et al. (2013). Similar results were also found in endophytic studies in *Cucumis sativus* and *Oryza sativa* (Mano et al. 2007; Mano and Morisaki 2008). The reason for such findings may be related with the bacterial population density in different tissues, which were estimated at ~10^6^ cells/g in leaf (Rastogi et al. 2012), while ~10^8^ cells/g in rhizosphere (Hardoim et al. 2008).

Jin et al. (2014) analyzed the distribution of endophytic bacteria in various tissues by 16S rRNA libraries and recorded that bacterial distribution may be associated with tissue specificity. Endophytic bacteria associated with the plant *Ferula songorica* during the current study were found to maintain both continuity and specificity in different tissue parts. A total of 13 genera were isolated in both root and ground tissues (leaf and stem), while the rest are specific to either of the two tissues.

There is increasing interest in developing potential biotechnological applications of endophytes for improving phytoremediation and sustainable production of non-food crops for biomass and biofuel production (Ryan et al. 2008). In a related study, Qin et al. (2014) have isolated ACC deaminase-producing endophytic bacteria from a halophytic plant *Limonium sinense* (Girard) Kuntze for evaluation of plant growth promotion under salt stress conditions. Strains belonging to genera *Bacillus*, *Pseudomonas*, *Klebsiella*, *Serratia*, *Arthrobacter*, *Streptomyces*, *Isoptericola* and *Microbacterium* have been shown to exhibit plant growth promoting traits such as phosphate solubilization, IAA production and ACC deaminase activity. During the present study, strains SZ4R6S2 and SZ4S4S9 showed high phosphate-solubilizing activities while few other strains have the ability to fix nitrogen and/or produce cellulase or protease. Strains Z4R7S10, SZ4R1S6 and SZ4S6S2 were positive for all the four activities tested. These results showed that the endophytic bacteria contain rich resources for use as biological control and biofertilizer.

Despite limitation in reflecting the true endophytic microbial diversity by culture-dependent studies (Nadkarni et al. 2009), it has its own advantages in that it gives an insight on the development and utilization of cultured microbial resources (Zhang 2005). Therefore, the use of culture-dependent method has a certain practical significance of reflecting diversity and distribution of endophytic bacteria and their functional role in plant ecological adaptation especially with special habitats such as in arid region of Xinjiang. Another disadvantage with the current method is that the distribution of the endophytes could be related with only the specific time and tissue of the individual plant and thus this method could not analyze the dynamic structure and distribution within the plant. Therefore, isolating the endophytes from different tissues and seasons, and combining them with rhizospheric microorganism is a practical method for providing better knowledge about the endophytic bacterial dynamic diversity.
